# Supplementary material for: Asynchronous magnetic resonance elastography: Shear wave speed reconstruction using noise correlation of incoherent waves
Source: Magn Reson Med. 2022 Oct 27;89(3):990–1001. doi: 10.1002/mrm.29502 (PMC9792433; doi:10.1002/mrm.29502)
Supplement: Supplementary file 1 — DATA S1 MATLAB code used to generate the numerical simulations of Figure 1 [file MRM-89-990-s002.zip › k-Wave/helpfiles/k-wave_examples.html]

Examples (k-Wave)


# Examples

Use this list to find examples in the documentation.

## Initial Value Problems

- Example: Homogenous Propagation Medium
- Example: Using A Binary Sensor Mask
- Example: Defining A Sensor Mask By Opposing Corners
- Example: Loading External Image Maps
- Example: Heterogeneous Propagation Medium
- Example: Saving Movie Files
- Example: Recording The Particle Velocity
- Example: Defining A Gaussian Sensor Frequency Response
- Example: Comparison Of Modelling Functions
- Example: Setting An Initial Pressure Gradient
- Example: Simulations In One Dimension
- Example: Simulations In Three Dimensions
- Example: Simulations In An Axisymmetric Coordinate System
- Example: Photoacoustic Waveforms In 1D, 2D and 3D

## Time Varying Source Problems

- Example: Monopole Point Source In A Homogeneous Propagation Medium
- Example: Dipole Point Source In A Homogeneous Propagation Medium
- Example: Simulating Transducer Field Patterns
- Example: Steering A Linear Array
- Example: Snell's Law And Critical Angle Reflection
- Example: The Doppler Effect
- Example: Diffraction Through A Slit
- Example: Simulations In Three Dimensions
- Example: Simulating CW Fields Using The Acoustic Field Propagator
- Example: Holographic Projections Using The Angular Spectrum Method
- Example: Equivalent Source Holography

## Sensor Directivity

- Example: Focussed Detector In 2D
- Example: Focussed Detector In 3D
- Example: Modelling Sensor Directivity In 2D
- Example: Modelling Sensor Directivity In 3D
- Example: Sensor Element Directivity In 2D
- Example: Focussed 2D Array With Directional Elements

## Photoacoustic Image Reconstruction

- Example: 2D FFT Reconstruction For A Line Sensor
- Example: 3D FFT Reconstruction For A Planar Sensor
- Example: 2D Time Reversal For A Line Sensor
- Example: 2D Time Reversal For A Circular Sensor
- Example: 3D Time Reversal For A Planar Sensor
- Example: 3D Time Reversal For A Spherical Sensor
- Example: Image Reconstruction With Directional Sensors
- Example: Image Reconstruction With Bandlimited Sensors
- Example: Attenuation Compensation Using Time Reversal
- Example: Attenuation Compensation Using Time Variant Filtering
- Example: Automatic Sound Speed Selection
- Example: Iterative Image Improvement Using Time Reversal
- Example: Iterative Image Reconstruction Using The Adjoint

## Diagnostic Ultrasound Simulation

- Example: Defining An Ultrasound Transducer
- Example: Simulating Ultrasound Beam Patterns
- Example: Using An Ultrasound Transducer As A Sensor
- Example: Simulating B-mode Ultrasound Images
- Example: Simulating B-mode Images Using A Phased Array

## Numerical Analysis

- Example: Controlling The Absorbing Boundary Layer
- Example: Source Smoothing
- Example: Filtering A Delta Function Input Signal
- Example: Modelling Power Law Absorption
- Example: Modelling Nonlinear Wave Propagation
- Example: Optimising k-Wave Performance

## Using The C++ Code

- Example: Running C++ Simulations
- Example: Saving Input Files in Parts

## Elastic Wave Propagation

- Example: Explosive Source In A Layered Medium
- Example: Plane Wave Absorption
- Example: Shear Waves And Critical Angle Reflection
- Example: Simulations In Three Dimensions

## Thermal Diffusion

- Example: Heat Diffusion In A Homogeneous Medium
- Example: Constant Rate Of Heat Deposition
- Example: Using A Binary Sensor Mask
- Example: Heating By A Focused Ultrasound Transducer
